# The Finite Element Simulation of the Upper Airway of Patients with Moderate and Severe Obstructive Sleep Apnea Hypopnea Syndrome

**DOI:** 10.1155/2017/7058519

**Published:** 2017-10-24

**Authors:** Huiping Luo, Austin Scholp, Jack J. Jiang

**Affiliations:** ^1^Department of Otolaryngology-Head and Neck Surgery, EENT Hospital of Fudan University, Shanghai, China; ^2^School of Medicine and Public Health, Department of Surgery, Division of Otolaryngology-Head and Neck Surgery, University of Wisconsin-Madison, Madison, WI, USA

## Abstract

**Objectives:**

To investigate the snoring modes of patients with Obstructive Sleep Apnea Hypopnea Syndrome and to discover the main sources of snoring in soft tissue vibrations.

**Methods:**

A three-dimensional finite element model was developed with SolidEdge to simulate the human upper airway. The inherent modal simulation was conducted to obtain the frequencies and the corresponding shapes of the soft tissue vibrations. The respiration process was simulated with the fluid-solid interaction method through ANSYS.

**Results:**

The first 6 orders of modal vibration were 12 Hz, 18 Hz, 21 Hz, 22 Hz, 36 Hz, and 39 Hz. Frequencies of modes 1, 2, 4, and 5 were from tongue vibrations. Frequencies of modes 3 and 6 were from soft palate vibrations. Steady pressure distribution and air distribution lines in the upper airway were shown clearly in the fluid-solid interaction simulation results.

**Conclusions:**

We were able to observe the vibrations of soft tissue and the modeled airflow by applying the finite element methods. Future studies could focus on improving the soft tissues vibration compliances by adjusting the model parameters. Additionally, more attention should be paid to vibrational components below 20 Hz when performing an acoustic analysis of human snore sounds due to the presence of these frequencies in this model.

## 1. Introduction

Obstructive Sleep Apnea Hypopnea Syndrome (OSAHS) is an upper respiratory disorder that has snoring as one of its symptoms. In order to obtain enough air while sleeping, a greater negative pressure is needed to pull air in through the upper airway. Consequently, the negative pressure causes a narrowing of the soft tissues. A positive feedback loop is created in this situation, when the airway narrows, more negative pressure is needed for air to be pulled through which again narrows the airway and eventually leads to collapse and snoring [1]. Another contributing factor to collapse is the weakening and loss of neural control in the upper airway, which is prominent in OSAHS patients [2]. Since the upper airway is essentially an elastic tube between the nasal cavity and the epiglottis, it is particularly susceptible to collapse under a normal negative pressure [2, 3]. Because snoring and airway collapse are a very common issue in OSAHS patients, much research has been done focusing on finding the site where the soft tissues will collapse. In previous studies, finite element (FE) simulation results revealed that the tongue base and the soft palate were parts that are most likely to collapse, which was consistent with clinical observations [3–6].

This paper focuses on the dynamic simulation of the upper airway model through SolidEdge and the ANSYS workbench. A modal simulation was performed to find the vibration shapes and frequencies of the model, and a combined fluid-solid interaction (FSI) with mechanics transient and computational fluid dynamic (CFD) was conducted to simulate the pressure in the airway.

## 2. Methods

### 2.1. The Design and Creation of a Three-Dimensional (3D) Model

**Figure 1:**
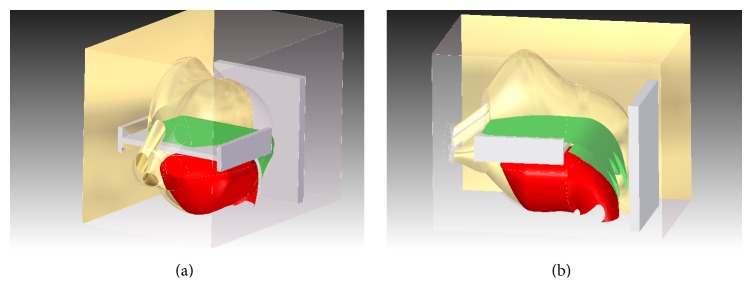
The 3D model in the SolidEdge work interface. (a) shows the anterior view where nares and the mouth were simplified as three holes. (b) is a lateral view. The green section is the palate, the red section represents the tongue body, and the white parts are bones.

To study the mechanism of the soft tissue vibration, we created a model to simulate the head and neck. Based on the anatomic atlas [7] and sagittal bitmaps of computed tomography (CT) scans acquired from OSAHS patients [6], a 3D model of the pharyngeal and nasal cavities was created in SolidEdge (Siemens PLM Software, Germany). In order to create a realistic anatomic structure while also facilitating the calculation of the finite element analysis (FEA), we designed a simplified 3D model. This model includes the tongue body, the hard palate, the soft palate, the uvula, the epiglottis, the posterior pharyngeal wall, and both lateral walls. The anterior wall of pharyngeal cavity is composed of the soft palate, the uvula, and the tongue. The air inlets, the mouth, and nostrils were simulated with three round holes. As shown in Figure 1, the green sections represented the soft palate and the red one is the tongue. Hard structures like the palate and the cervical vertebra were simulated with fixed white flake structures. Due to the limitations of geometric simulation software requirements, some features needed to be simplified. For this reason, we did not model the lower upper airway and simplified the head and neck into solid or platy structures. All parameters such as diameter and cross-sectional area that are used to establish the model were similar to those in previous research [6]. The finished model (Figure 1) was imported into ANSYS (R15.0, Pittsburgh, PA, USA). Then the modal analysis was conducted by the Modal ANSYS module in ANSYS workbench. A pressure simulation was done together with transient structure module and ANSYS CFX. The interface between these two modules was the airflow and the wall composed of the pharyngeal soft tissues.

### 2.2. The Choice of Materials Properties and Boundary Conditions

**Figure 2:**
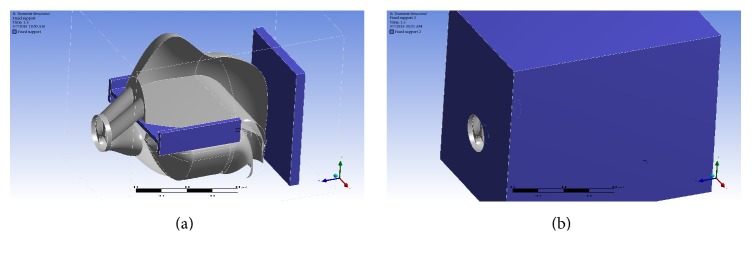
The 3D model after importing to the ANSYS workbench. The grey structures show the soft tissues of the nasal cavity, tongue, and soft palate. The blue represents bony structures. In (b), the rest of the skull is simplified to a box of hard tissue.

The human pharyngeal cavity was considered to be mechanical system which could be mathematically described through the theories of solid and fluid mechanics. Our model was deigned according to previous studies' mathematical descriptions [4, 5] which were shown in Table 1. We set bony structures to a fixed position. To prevent modes of other parts like the face, the head, and the neck (Figure 2) from occurring, they were set as fixed as well. For the pressure simulation, the pressure difference between the model entrances and the glottis was set to be −20 Pa in accordance with Van Holsbeke et al. [8].

## 3. Results

### 3.1. Modal Simulation Analysis

**Figure 3:**
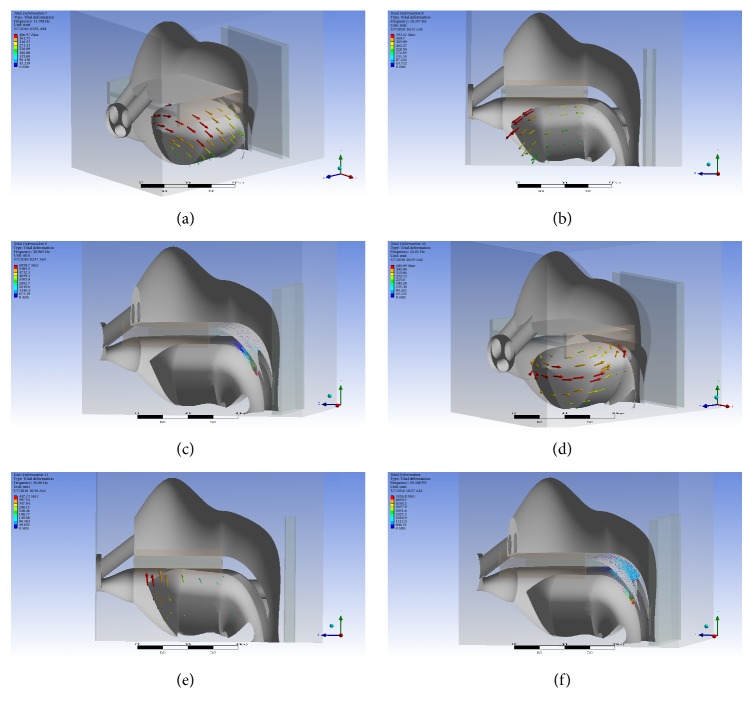
Simulated modes 1–6 are shown here in images (a)–(f). In modes 1, 2, 4, and 5, the tongue vibration can be seen with displacements in different directions. In modes 3 and 6, soft palate vibrations are observed. In mode 6, there are significantly more vibration seen at the uvula.

Frequencies of first 6 orders (Figure 3) corresponding to the soft tissue vibrations were 12 Hz, 18 Hz, 21 Hz, 22 Hz, 36 Hz, and 39 Hz. The results from the modal simulation are outlined in Table 2. Modes 1, 2, 4, and 5 were found to be vibrations of the tongue with frequencies that ranged from 11 to 30 Hz. Modes 3 and 6 were vibrations of the soft palate with frequencies of 21 Hz and 39 Hz. Each mode had a dominant axis of rotation, which is also outlined in the table. Mode 1 showed to have dominant displacements around the *x*-axis. Modes 2 and 3 were dominant around the *z*-axis while modes 4 and 5 were dominant around the *y*-axis. Mode 6 was a combined vibration of the soft palate and the uvula. It showed a 2nd-order distortion, which has too complicated of a movement to describe. In reality, the snoring of the patient is the combination of these vibrations.

### 3.2. Pressure Simulation

**Figure 4:**
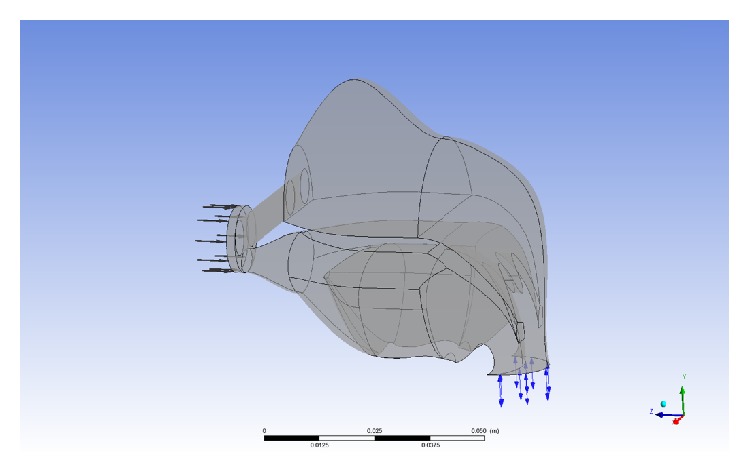
The input and output direction of the fluid-structure coupling analysis in ANSYS workbench.

**Figure 5:**
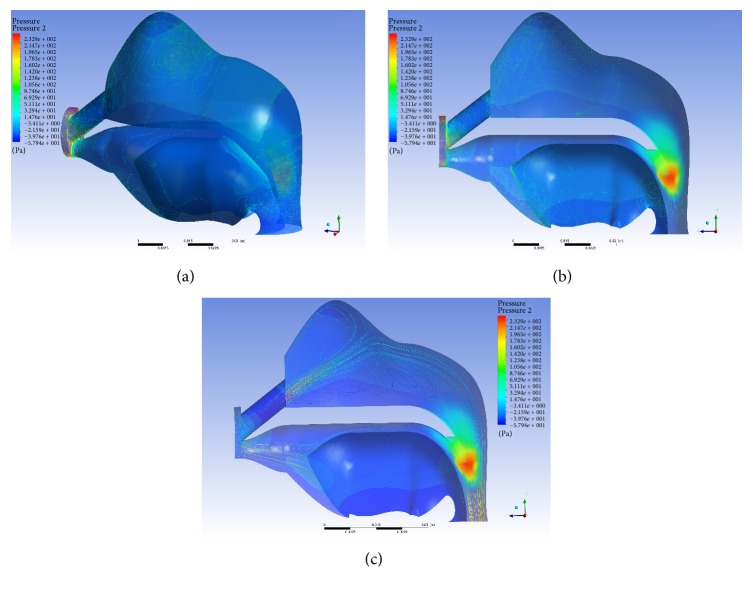
ANSYS stress and flow simulation results. These were obtained from the FSI method. (a) The stress distribution under a constant pressure. (b) Under simulated breathing, an analysis of the maximum stresses reveals that the point of highest stress is located in the soft palate at the tip of the uvula. (c) Adding flow lines shows the general airflow through the upper airway in simulated breathing.

The results of the fluid-structure coupling simulation are shown in Figures 4 and 5. Here we can see the steady pressure distribution and distribution lines. The pressure distributed mostly in nasal cavity and pharyngeal cavity (Figures 5(a) and 5(b)). The airflow and pressure concentrated on the palatopharynx and glossopharynx, while the maximum velocity occurred on the tip of uvula (Figure 5(c)).

## 4. Discussion

The production of snoring involves a composite of various soft tissue vibrations and dynamic changes of anatomic structures in the upper airway. Vibrations of upper airway tissues may affect the airway pressure and result in the opening and closing of soft tissue flaps. Acoustic parameters of snore sounds are derived from the interaction of biomechanical properties and the neuromuscular activity of the upper airway which makes the simulation of snore sounds production even more complicated [9–11]. Due to the convergence in the FE simulation, models need to be simplified. To get closer to a more realistic simulation, researchers have been continuously trying to establish a model which has a more accurate physiological morphology.

The pressure analysis done here illustrates a change during nocturnal breathing coupled with a deformation of soft tissues in the oral and pharyngeal cavities. This helps researchers obtain a better understanding of the pathological mechanism of snore sound production. In the dynamic response analysis of FEA, material properties of the soft palate and the tongue were assumed to be viscoelastic materials, whose movements were regarded as nonlinear [12–14]. Since sources of snore sound production were soft tissue vibrations of different segments, vibration frequencies were able to be obtained from modal simulation analysis. Liu et al.'s study showed that vibration frequencies of the head tissues were in the range of 10 to 60 Hz. These were concentrated in the nose, the soft palate, and the tongue area. The lowest frequency was 14.4 Hz, and the main deformation was concentrated in the nasal cavity and the soft palate. The most sensitive area was the free edge of the soft palate, whose frequency was 22.4 Hz. The only response frequency of the tongue body was 22.6 Hz [4]. Our results showed that the 11.798 Hz vibration began in tongue body vibration, and the palate vibration began in 20.965 Hz, which were similar to the results of Liu et al. [4]. Compared with the 22.6 Hz vibration in Liu et al.'s study, which mainly resulted from tongue vibration, it may be derived from the tongue body vibration of modal 4 in modal simulation. Comparing this with acoustic analysis results of realistic snore sounds, it may help in diagnosing the main vibration anatomic structures and obstructive sites.

According to simulation results, frequencies of first 4 orders were in the range from 11.798 to 22.01 Hz, which is in the range of infrasonic frequencies. Sounds in this range are not audible to human ears, and there has not be any acoustic analysis research focusing on infrasonic components of human snore sounds. In an unpublished study of the acoustic analysis of snore sounds in our lab, the average frequencies of the first to the fifth peaks in the DFT spectrums were found to be 12.5 Hz, 17.3 Hz, 19.3 Hz, 33.2 Hz, and 43.2 Hz. These acoustic results verify our simulation results in a certain degree.

Certain anatomical structures could have direct effects on snoring. Other factors, such as sleep postures, sleep stages, and breathing condition, could affect snore sound production and sound quality [15]. Therefore, we may need to consider these factors in future research. By changing parameters of the 3D model and simulating airways of OSAHS patients, comparing the results with normal airways as a control, obtaining acoustic parameters of benign snoring and acoustic parameters, we can update our model and produce new FEA results. Through continuous efforts, we believe that we can obtain a better understanding of the pathophysiology of snoring production by doing FE simulations of the 3D upper airway model and eventually develop new tools for OSAHS diagnosis based on snore detection and obstructive sites positioning. Furthermore, to verify the specificity and sensitivity of the method by comparing simulation results with the acoustic analysis of clinical data, hopefully we can develop finite element methods (FEM) as a standard for OSAHS diagnosis, guiding the selection of therapeutic methods and evaluating the efficacy of surgical treatments in treating OSAHS.

## 5. Conclusions

FEA can be used to simulate vibrations of the soft palate and the tongue. Together with CFD and mechanical analysis, it can illustrate pressure distribution and airflow-soft tissue interactions. With adjustment of model parameters based on anatomical data, researchers could obtain more accurate simulation results. Acoustic analysis of human snore sounds should be done to verify whether simulation results conform to the actual situation. The results of our simulation and acoustic analysis suggest that more attention needs to be paid to frequencies below 40 Hz, especially the infrasonic components.

## Figures and Tables

**Table 1:** Material properties of the FE model used in the simulation.

Material	Young's modulus *E* (MPa)	Poisson's ratio (*μ*)	Density *ρ* (kg/m^3^)
Hard palate	2	0.22	1040
Soft palate	0.025	0.42	1040
Tongue	0.015	0.499	1040

**Table 2:** The vibration frequency and location for each mode.

Mode	Frequency (Hz)	Location
1 (a)	12	Tongue
2 (b)	18	Tongue
3 (c)	21	Soft palate
4 (d)	22	Tongue
5 (e)	36	Tongue
6 (f)	39	Soft palate
